# Epoxy-Coated Side-Polished Fiber-Optic Temperature Sensor for Cryogenic Conditions

**DOI:** 10.3390/s23052850

**Published:** 2023-03-06

**Authors:** Umesh Sampath, Minho Song

**Affiliations:** 1 Department of Electronics and Information Engineering, Jeonbuk National University, Jeonju 54896, Republic of Korea; 2 Electronic & Control Group, INZI Controls Co., Ltd., Siheung 15090, Republic of Korea

**Keywords:** side-polished optical fiber, optical sensor, epoxy, cryogenic temperature, refractive index

## Abstract

We propose coating side-polished optical fiber (SPF) with epoxy polymer to form a fiber-optic sensor for cryogenic temperature measuring applications. The thermo-optic effect of the epoxy polymer coating layer enhances the interaction between the SPF evanescent field and surrounding medium, considerably improving the temperature sensitivity and robustness of the sensor head in a very low-temperature environment. In tests, due to the evanescent field–polymer coating interlinkage, transmitted optical intensity variation of 5 dB and an average sensitivity of −0.024 dB/K were obtained in the 90–298 K range.

## 1. Introduction

For decades, efforts have been made to develop optical fiber sensors for a broad range of chemosensing, biosensing, structural health monitoring applications, as well as physical properties detection. Optical fiber sensors have certain advantages over conventional electrical sensors, such as electromagnetic interference immunity, corrosion resistance, and fast response, which make them excellent candidates for various types of measurement applications, including temperature sensing [1].

Among optical fibers, side-polished optical fibers (SPFs) are utilized extensively in the field of optimal design methods and sensing applications based on the tunability of the effective index parameter and the energy distribution in the optical fiber transmission. The SPF sensors function by monitoring the interactions between the evanescent waves and surrounding medium. When the SPF is utilized for an optical sensor, this is considered to be the Kretchmann’s configuration [2]. The SPF sensor is realized by depositing the analyte on the SPF area, and it can be a functional material that is sensitive to a change in external conditions such as temperature, pH, magnetic field, and humidity [3,4]. Many SPF sensor schemes have been employed to measure ambient temperatures. He et al. [5] have tested an all-fiber temperature sensor on coreless SPF wrapped with polydimethylsiloxane and reported high sensitivity of −0.4409 nm/°C in the temperature range of 30–85 °C. Lu et al. [6] have lowered the temperature range down to −7 °C by depositing TiO_2_ nanoparticles on SPFs. Although all the techniques mentioned above can be successfully used in the environments around room temperature, certain complex applications such as superconducting magnets and wires, aerospace, and biological experiments may demand fiber-optic sensors with cryogenic temperature monitoring capability [7]. To the best of our knowledge, the SPF sensor working under the cryogenic condition has not been reported. Usually, the fiber Bragg grating (FBG) has been employed to achieve cryogenic temperature measurement [8,9,10]. Chiuchiolo et al. [9] have measured the cryogenic temperature with enough sensitivity by using an FBG sensor coated with epoxy and PMMA. Additionally, Lupi et al. [8] and Rajini et al. [10] have improved the sensitivity of the FBG sensor at cryogenic temperatures by depositing various metals on aluminum pre-coated bare FBG. However, due to the increases in cost and the complexity of the design, alternative research efforts have focused on developing simple sensor structure and cost-effective demodulation techniques [11,12].

In this study, we demonstrate a simple and cost-effective evanescent field-based optical platform for cryogenic temperature sensing. An SPF is coated with epoxy that has a large thermo-optic coefficient and the coated layer is used as a sensor head for very low temperature monitoring. The strong evanescent light of the SPF can couple with the interface between the epoxy and the polished fiber surface, which enhances the interaction between light and the surrounding medium, resulting in stronger sensitivity to the external temperature change. The proposed epoxy-coated SPF sensor is a transmission-type sensor: the temperature changes in surrounding medium can be traced by measuring the optical power passing though the sensor head. We conducted a series of experiments testing the feasibility, the repeatability, and the robustness of the proposed epoxy-coated SPF sensor in temperature range of 90 to 298 K.

## 2. Principle and Device Fabrication

Light propagations in the optical fiber depend on the principle of attenuated total reflection (ATR) [13]. The light propagating along the fiber core strikes the core–cladding interface at an angle θ greater than the critical angle, which results in a total internal reflection. However, only small amounts of light energy escape into the cladding medium and it generates its own electromagnetic field in the cladding or surrounding medium, which is known as an evanescent wave (EW) [14].

Absorption of EW into the surrounding medium of the fiber leads to rapid attenuation of the light, which was explained by the theory of ATR. The lights in a de-cladded fiber section interact with the surrounding medium, which is the material under investigation. This interaction modulates or attenuates the light signal due to a change in the refractive index of the material under investigation [15].

**Figure 1:**
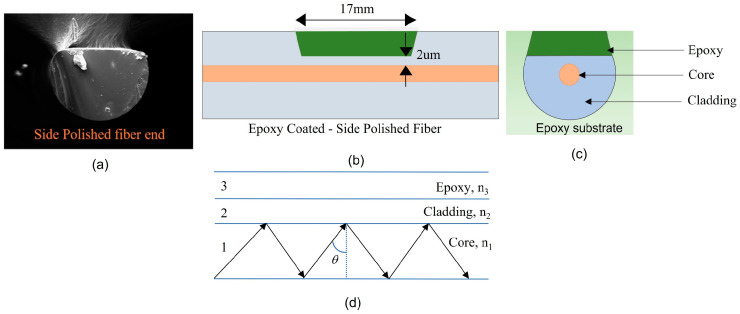
Side-polished optical fiber (SPF) (**a**) SEM cross-section image of SPF, (**b**) schematic diagram of SPF with epoxy coating, (**c**) schematic cross-sectional view of coated SPF, (**d**) refractive index: core, cladding, and epoxy.

We used a single-mode fiber (SMF28) with effective core and cladding dimensions of ~10 and 125 µm, respectively, to fabricate SPFs. The epoxy substrate was used as mechanical support and the material was the same as the epoxy coating material. The optical fiber was embedded in the substrate and polished until the cladding remained only few microns from the core. A matured wheel side-polishing technique [16] was used for polishing, and the total length of the polished region was 17 mm. The insertion loss with ambient air condition was measured to be less than 0.1 dB. Figure 1a is the SEM (scanning electron microscope) image of the SPF’s cross-section, which we constructed later using a modified cladding with epoxy resin. The polymer base used for the cladding was bisphenol-A diglycidyl ether, and the curing agent was 4, 4′ diamino-diphenylmethane. Figure 1b–d shows the basic structure of the SPF sensor head with modified cladding, the SPF sensor’s cross-sectional schematic diagram, and the refractive index distribution of the core, cladding, and epoxy resin, noted as $\;n_{1}$, $n_{2}$, and $n_{3}$, respectively.

The primary criteria of SPF-based temperature sensors are the changes in the refractive index of the material against the applied temperature changes. Additionally, the model for effective evanescent wave sensor reveals that the variation in the refractive index of coating material should be greater than the changes in the refractive indices of cladding and core, which are mostly silica glass (SiO_2_). Considering our previously reported results from the Fresnel reflection-based cryogenic temperature sensor, we prepared the SPF sensors with an epoxy polymer coating. The thermo-optic coefficient of epoxy is $dn/dT=-1.3\times 10^{-4}/{}^{\circ}C$ at room temperature [11,16]. Because silica glass has $dn/dT=9.2\times 10^{-6}/{}^{\circ}C$ that is approximately one order of magnitude less than the polymer’s, most of the change in transmission intensity is due to the variation in the refractive index of the epoxy polymer.

The thermo-optic coefficient of polymer is theoretically modeled as follows:

(1) $$\frac{dn}{dT}=\frac{\left(n^{2}-1\right)\left(n^{2}+2\right)}{6n}\left(\Phi -\beta \right)$$

where Φ is the temperature coefficient of the electronic polarizability and β is the thermal expansion coefficient [17]. The value is not constant over a wide temperature range because density and electronic polarizability change with temperature, resulting in a relationship between transmission light intensity and temperature change. In epoxy, dn/dT is negative and decreases with increasing temperature because their β are always much higher than their polarizability Φ [11].

## 3. Experiments and Results

**Figure 2:**
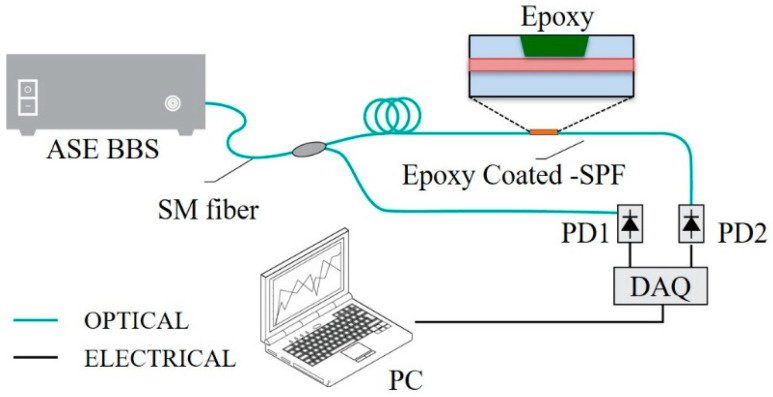
Experimental setup of the proposed fiber-optic sensor system. SM—single mode; SPF—side-polished fiber; PD—photo detector; DAQ—data acquisition; ASE BBS—amplified spontaneous emission broadband source.

To test the epoxy-coated-SPF sensor in cryogenic temperature detection, we constructed a fiber-optic sensor system (Figure 2). As a light source, we used a C-band ASE (amplified spontaneous emission) BBS (broadband light source), which had a center wavelength and output power of 1550 nm and 12 dBm, respectively. The light passed though the sensor and the transmitted light was collected by photodetector PD2. The transmittance was determined based on the temperature-dependent refractive index difference at the sensing layer of the coated SPF sensor. To minimize errors resulting from possible fluctuations in the light-source power and transmission losses in the fiber-optic links, a fiber-optic directional coupler was used to tap a certain portion of light and its power was measured with PD21 and used for referencing.

The fabricated epoxy-coated SPF was placed in a temperature chamber, and the temperature variation was monitored by a reference thermocouple as well as the proposed fiber-optic sensor. By pouring liquid nitrogen (LN_2_) and waiting for the natural evaporation process, the temperature measurements were conducted in the range of 90–298 K.

**Figure 3:**
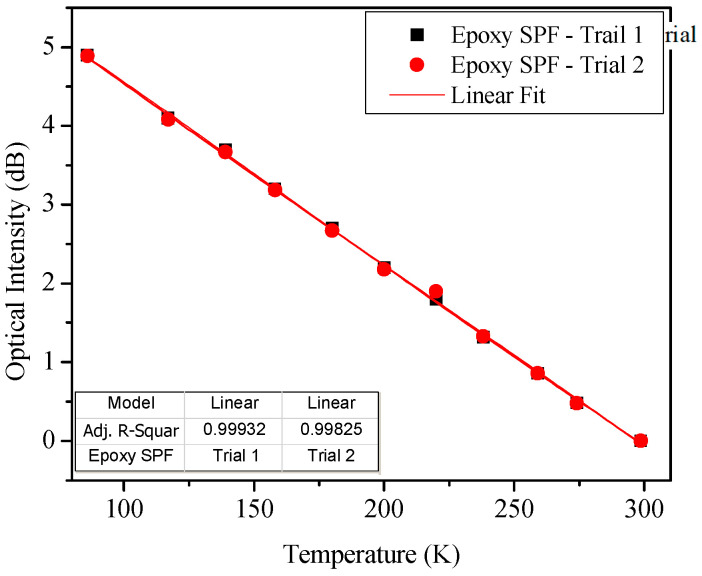
Cryogenic temperature measurements with epoxy-coated SPF sensor. The output is optical intensity variation according to temperature change in 90–298 K.

Figure 3 shows the results of the tests. As the temperature increased from 90 K, the cryogenic temperature of liquid nitrogen, the transmitted light intensity decreased in linear fashion by as much as 5 dB for the whole measurement range. Almost the same results were obtained in the subsequent measurement cycles. From the measured data and their linear regression, the average temperature sensitivity and R^2^ value were −0.024 dB/K and 0.999, respectively.

For additional feasibility test, we compared the sensor result with those of commercially available high-resolution T-type reference thermocouple (73–623 K) and a customized FBG sensor that was proposed in the earlier report [7]. The low thermal response of FBG in cryogenic temperature conditions was overcome by the polymer coated-FBG (PCFBG). The average temperature sensitivity of the PCFBG sensor was 48 pm/K in the range of 92–298 K, and the reflected Bragg wavelength was demodulated by a spectrometer (FBGA, Bayspec), which had a wavelength readout resolution of 1 pm.

**Figure 4:**
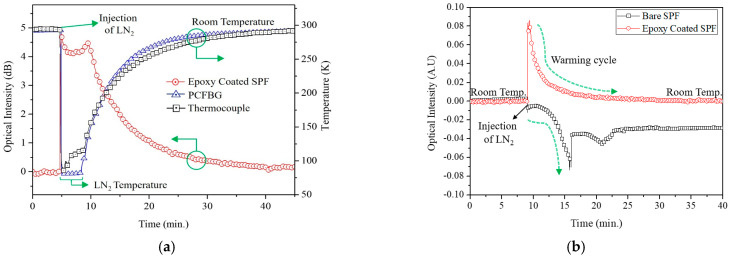
Performance comparison of the proposed sensor: (**a**) Bare SPF sensor vs. epoxy-coated SPF sensor; (**b**) reference sensors (PCFBG and T-type thermocouple) vs. epoxy-coated SPF sensor.

The sensors were placed in the temperature chamber at room temperature and then liquid nitrogen was poured. As the LN_2_ evaporated, the temperature slowly increased and reached initial room temperature in 40 min. In Figure 4a, the measured outputs of the reference sensors (PCFBG and thermocouple) mostly agreed well in the whole range. Additionally, the output of the proposed epoxy-coated SPF matched with the reference sensors’ outputs, although it was not calibrated in the temperature unit and changed in the negative direction due to the epoxy’s negative thermo-optic coefficient.

In general, an intrinsic, bare SPF is very sensitive to changes in the external environment. A sensitive reaction, however, does not necessarily indicate that the sensor is good. To demonstrate the advantage of epoxy coating, a bare SPF was employed with the same measurements as the epoxy-coated one. Figure 4b shows the measurement results of the different SPFs. The epoxy-coated SPF sensor with the substrate presented full function of temperature sensing from the sharp output increase at LN_2_ injection until it finally reached the original output at room temperature. The bare SPF, however, was disrupted and the output fluctuated in the middle of warming cycle and never regained its original position at room temperature. This could be attributed to the fragile and weak structure with only 2 μm of cladding thickness.

**Figure 5:**
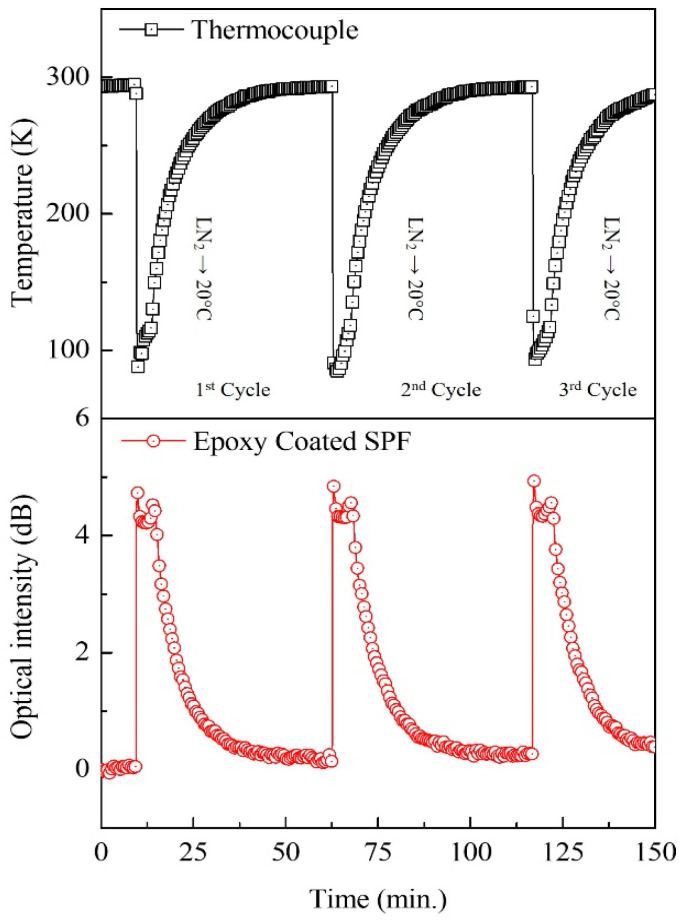
Repeatability test in multiple measurement cycles. Upper trace: thermocouple output; lower trace: output from the epoxy-coated SPF sensor.

Repeatability is one of the most desirable traits of sensors, especially in harsh environments. By applying repeated cycles of LN_2_ injection and evaporation, we tested the repeatability of the epoxy-coated SPF sensor. Based on the measurement results of three cycles (lower trance in Figure 5), no significant deviations from the other cycles could be found, and a thermocouple output (upper trace in Figure 5) was successfully used as a reference. No noticeable damage to the epoxy-coated SPF sensor was found, even after multiple cycles of cryogenic temperature measurements.

## 4. Conclusions

In conclusion, we have successfully demonstrated that an epoxy-coated side-polished fiber could be used in cryogenic temperature monitoring. Due to the surface evanescent field enhancement along the side-polished waveguide resulting from the epoxy polymer coating, sensitivity of –0.024 dB/K, good repeatability, and a robust and stable sensing capability in a very low temperature range, 90–298 K, could be obtained. Considering the simple structure, the low cost, the easy demodulation processing, and the fast response, the proposed epoxy-coated SPF sensor is an excellent candidate sensor, with inherent dielectric nature, for cryogenic temperature sensing applications. By further optimizing the structure of the SPF sensor, for example, by decreasing the coating thickness and/or varying the coating material, a higher sensitivity might be achieved, and the temperature range could be extended.

## Data Availability

Data sharing is not applicable to this article.

## References

[1] Sampath U., Kim H., Kim D., Kim Y., Song M. (2015). In-situ cure monitoring of wind turbine blades by using fiber Bragg grating sensors and Fresnel reflection measurement. Sensors.

[2] Ying Y., Si G., Luan F., Xu K., Qi Y., Li H. (2016). Recent research progress of optical fiber sensors based on D-shaped structure. Opt. Laser Technol..

[3] Botewad S.N., Pahurkar V.G., Muley G.G. (2016). Fabrication and evaluation of evanescent wave absorption-based polyaniline-cladding modified fiber optic urea biosensor. Opt. Fiber Technol..

[4] Jung W., Kim S., Kim K., Kim E., Kang S. (2001). High-Sensitivity Temperature Sensor Using a Side-Polished Single-Mode Fiber Covered with the Polymer Planar Waveguide. IEEE Photonic. Technol. Lett..

[5] He C., Fang J., Zhang Y., Yang Y., Yu J., Zhang J., Guan H., Qiu W., Wu P., Dong J. (2018). High performance all-fiber temperature sensor based on coreless side-polished fiber wrapped with polydimethylsiloxane. Opt. Exp..

[6] Lu H., Tian Z., Yu H., Yang B., Jing G., Liao G., Zhang J., Yu J., Tang J., Luo Y. (2014). Optical fiber with nanostructured cladding of TiO2 nanoparticles self-assembled onto a side polished fiber and its temperature sensing. Opt. Exp..

[7] Sampath U., Kim D., Kim H., Song M. (2018). Polymer-coated FBG sensor for simultaneous temperature and strain monitoring in composite materials under cryogenic conditions. Appl. Opt..

[8] Lupi C., Felli F., Brotzu A., Caponero M.A., Paolozzi A. (2008). Improving FBG sensor sensitivity at cryogenic temperature by metal coating. IEEE Sens. J..

[9] Chiuchiolo A., Palmieri L., Consales M., Giordano M. (2015). Cryogenic-temperature profiling of high-power superconducting lines using local and distributed optical-fiber sensors. Opt. Lett..

[10] Rajini-Kumar R., Suesser M., Narayankhedkar G., Krieg G., Atrey M.D. (2008). Performance evaluation of metal-coated fiber Bragg grating sensors for sensing cryogenic temperature. Cryogenics.

[11] Sampath U., Kim D.G., Kim H., Song M. (2018). Cryogenic temperature sensor based on Fresnel reflection from a polymer-coated facet of optical fiber. IEEE Sens. J..

[12] Sampath U., Kim D.G., Song M. Side-Polished Fiber-Optic Temperature Sensor for Cryogenic Conditions. Proceedings of the 26th Conference on Optical Fiber Sensors.

[13] Paul P.H., Kychakoff G. (1987). Fiber-optic evanescent field absorption sensor. Appl. Phys. Lett..

[14] Memon S.F., Ali M.M., Pembroke J.T., Chowdhry B.S., Lewis E. (2017). Measurement of ultralow level bioethanol concentration for production using evanescent wave based optical fiber sensor. IEEE Trans. Instrum. Meas..

[15] Sharma A.K., Gupta J., Sharma I. (2019). Fiber-optic evanescent wave absorption-based sensors: A detailed review of advancements in the last decade (2007–2018). Optik.

[16] Tang J., Zhou J., Guan J., Long S., Yu J., Guan H., Lu H., Luo Y., Zhang J., Chen Z. (2017). Fabrication of Side-Polished Single Mode-Multimode-Single Mode fiber and Its Characteristics of Refractive Index Sensing. IEEE J. Sel. Top. Quant..

[17] Kang E.S., Lee T.H., Bae B.S. (2002). Measurement of the thermos-optic coefficients in sol-gel derived inorganic-organic hybrid material films. Appl. Phys. Lett..

